# Expression and strain variation of the novel “*small open reading frame*” (*smorf*) multigene family in *Babesia bovis*

**DOI:** 10.1016/j.ijpara.2011.10.004

**Published:** 2012-02

**Authors:** Lucas M. Ferreri, Kelly A. Brayton, Kerry S. Sondgeroth, Audrey O.T. Lau, Carlos E. Suarez, Terry F. McElwain

**Affiliations:** a Department of Veterinary Microbiology and Pathology and School for Global Animal Health, College of Veterinary Medicine, Washington State University, Pullman, WA 99164-6040, USA; b Animal Disease Research Unit, United States Department of Agriculture, Agricultural Research Service, Pullman, WA 99164, USA

**Keywords:** *Babesia bovis*, *Smorf* multigene family

## Abstract

*Small open reading frame* (*smorf*) genes comprise the second largest *Babesia bovis* multigene family. All known 44 variant *smorf* genes are located in close chromosomal proximity to *ves1* genes, which encode proteins that mediate cytoadhesion and contribute to immune evasion. In this study, we characterised the general topology of *smorf* genes and investigated the gene repertoire, transcriptional profile and SMORF expression in two distinct strains, T2Bo and Mo7. Sequence analysis using degenerate primers identified additional *smorf* genes in each strain and demonstrated that the *smorf* gene repertoire varies between strains, with conserved and unique genes in both. *Smorf* genes have multiple semi-conserved and variable blocks, and a large hypervariable insertion in 20 of the 44 genes defines two major branches of the family, termed *smorf* A and *smorf* B. A total of 32 *smorf* genes are simultaneously transcribed in T2Bo strain *B. bovis* merozoites obtained from deep brain tissue of an acutely infected animal. SMORF peptide-specific antiserum bound in immunoblots to multiple proteins with a range of sizes predicted by *smorf* genes, confirming translation of *smorf* gene products from these transcripts. These results indicate that the *smorf* multigene family is larger than previously described and demonstrate that *smorf* genes are expressed and are undergoing variation, both within strains and in a lineage-specific pattern independent of strain specificity. The function of these novel proteins is unknown.

## Graphical abstract

**Graphical abstract:**

**Figure:**
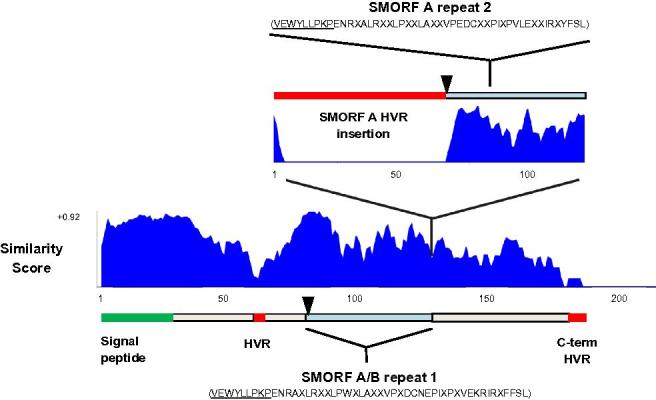

**Highlights:**

► The *smorf* gene family is the second largest in *Babesia bovis*. ► *Smorf* genes have a large hypervariable insertion in 20 of the 44 genes. ► The *smorf* gene repertoire varies between strains, with both conserved and unique genes in each strain. ► Multiple *smorf* genes are transcribed and expressed in merozoites.

## 1 Introduction

Multigene families play a key role in the biology and persistence of pathogens. Examples of multigene families include the *var*, *rifin*, *stevor* and *Pfmc-2TM* multigene families in *Plasmodium falciparum* (Sam-Yellowe et al., 2004; Kyes et al., 2007; Blythe et al., 2008; Bultrini et al., 2009), *svsp* and *tpr* in *Theileria parva* and *ves1* in *Babesia bovis* (Allred et al., 2000). In *Plasmodium* and *Babesia* spp., the *var* and *ves1* gene products, *P. falciparum* erythrocyte membrane protein 1 (PfEMP1) and *B. bovis* variant erythrocyte surface antigen 1 (VESA1), respectively, are exported to the surface of infected erythrocytes, where they mediate cytoadhesion to endothelial cells in multiple tissues and undergo antigenic variation, allowing persistence within the mammalian host (Smith et al., 1995; O’Connor and Allred, 2000). *Plasmodium falciparum rifin* and *stevor* multigene families are positionally associated with *var* genes, but the function of STEVOR and RIFIN proteins has been elusive. Similarly, the function of *Theileria* TPR and SVSP proteins is unknown.

A *B. bovis*-specific novel multigene family termed *small open reading frame* or “*smorf*” was recently identified in the *B. bovis* genome, and with 44 members is the second largest multigene family described in *B. bovis* (Brayton et al., 2007). Members of this gene family do not have significant sequence identity with any other gene or protein in available databases. Similar to the physical proximity of *var*, *rifin* and *stevor* genes in the *P. falciparum* genome, *B. bovis smorf* genes are always found within 4 kb of members of the *ves1* multigene family. However, unlike the primarily subtelomeric location of many plasmodial multigene families, *ves1* and *smorf* genes are distributed throughout all four *B. bovis* chromosomes. The 44 *smorf* genes range in length from 327 to 1,377 nucleotides, with a degree of conservation between 28% and 95%, and often occur in pairs, oriented in both a head to head and head to tail arrangement (Brayton et al., 2007).

Beyond an initial analysis showing multiple *smorf* transcripts in the uncloned *B. bovis* T2Bo strain (Brayton et al., 2007), little is known about these novel genes or their gene products. In this study, we characterised the topology of the *smorf* gene family, examined strain variation and investigated their transcriptional profile and protein expression in both cloned and uncloned parasite strains.

## 2 Materials and methods

### 2.1 Parasite strains, culture and cloning

The Mo7 clone of *B. bovis* was derived by limiting dilution of a Mexican strain as described elsewhere (Hines et al., 1989). The parental strain was originally isolated from northern Mexico. Parasites were grown in long-term microaerophilus stationary-phase culture in bovine erythrocytes (Levy and Ristic, 1980). The Mo7 clonal line was re-cloned by limiting dilution for analysis of *smorf* transcription and translation, and the newly derived clone was designated Mo7.2. The T2Bo strain of *B. bovis*, originally isolated from Texas near the Mexican border, has also been described (Goff et al., 1998), and was used for derivation of the genome sequence from which the *smorf* multigene family was originally identified.

### 2.2 Transcriptional and sequence analysis

#### 2.2.1 Genomic DNA (gDNA) isolation

Genomic DNA was purified from *B. bovis* cultured parasites and T2Bo strain-infected bovine brain tissue using TRIzol reagent (Invitrogen, USA) following manufacturer’s specifications. The T2Bo-infected brain tissue was obtained from a spleen-intact calf infected i.v. with T2Bo liquid nitrogen preserved stabilate. The course of infection in this experimentally infected animal has been described (Bastos et al., 2010). The calf demonstrated classical clinical signs of acute babesiosis referable to elevated body temperature (∼40 °C), decreased haematocrit as a result of erythrocyte haemolysis (packed cell volume (PCV) 12, a decrease of 59% from pre-infection), and depression, recumbency and abnormal mentation attributable to anaemia and neurological disease. It died at day 15 p.i. Brain tissue was collected into 10% buffered neutral formalin or stored in liquid nitrogen for gDNA and RNA isolation. Frozen, unfixed T2Bo-infected brain samples were thawed and disrupted in Lysis Matrix D tubes (MP Biomedicals Inc., USA) run on a homogeniser (MP Biomedicals Inc., FastPrep-24) at 4.0 m/s, and gDNA isolated using the TRIzol protocol.

#### 2.2.2 T2Bo smorf primers

Specific primers were designed to amplify unique regions of each *smorf* gene (Brayton et al., 2007) (Supplementary Table S1). In order to confirm their specificity, each amplicon was cloned into a pCR2.1 plasmid vector (Invitrogen) and sequenced. TheT2Bo *smorf* genes amplified by each primer set are indicated in Supplementary Table S1.

#### 2.2.3 cDNA and specific smorf amplifications

Total RNA was isolated from cultured and brain tissue-derived parasites using TRIzol following the manufacturer’s protocol. Twenty-five μg of total RNA were treated twice with 2 U of DNase at 37 °C for 30 min, each followed by 5 μl of DNase inactivator at room temperature for 2 min. Following centrifugation at 10,000*g* for 1.5 min, the supernatant was transferred to a clean tube and stored at −80 °C until use. cDNA was obtained from total RNA using Random Decamers from the Retro Script kit (Ambion, USA following the manufacturer’s instructions. A negative control containing total RNA and no reverse transcription (RT) was produced in parallel. PCR amplification was carried out using as template cDNA, no-RT treated RNA and gDNA by denaturing at 95 °C for 4 min, followed by 40 cycles of 1 min at 95 °C, 1 min at the specific primer set annealing temperature and 1.5 min at 72 °C, with a final extension step of 10 min at 72 °C.

#### 2.2.4 Amplification and sequencing of the Mo7 smorf gene family

In order to characterise new *smorf* genes in the Mo7 *B. bovis* strain, degenerate primers were designed from sequences within the open reading frame (ORF) and in the 5′ and 3′ flanking regions of T2Bo *smorf* gene members. Two forward degenerate primers: F1-5′ ATG GTA GCC TTY AAC ACH TT 3′, and F2-5′ CAC TCY YTA CTG ACT TYA CCA 3′; and three reverse degenerate primers: R1-5′ KGG TCT ARC GDG ACT AAT ATT GAT 3′, R2-5′ GTC ATR ATG ATT CAT KTA TAG AGA TGC 3′, and R3-5′ CCA TGG GAC AYT ATG GAT ACC 3′, all with a melting temperature of approximately 55 °C, were synthesised. Amplification conditions were the same as those using T2Bo-specific *smorf* primers except with a longer extension step of 2 min. Amplification products were cloned in a pCR2.1 vector (Invitrogen) following the manufacturer’s instructions. Colonies were grown in 96 well culture plates for 16 h using 2YT medium (16 g of tryptone, 10 g of yeast extract and 10 g of sodium chloride in 1 L) with 100 μg/ml of carbenicillin. Bacterial cultures were processed using a Manual Perfectprep™ Plasmid 96 Vac Kit (5Prime). Sequencing was carried out using the BigDye Terminator v3.1 Cycle Sequencing Kit (Applied Biosystems), using either M13 forward or reverse primers. The sequencing program was 96 °C for 1 min, followed by 25 cycles of 96 °C for 10 s, 50 °C for 5 s, 60 °C for 4 min, and a final extension for 7 min at 60 °C.

### 2.3 Immunodetection of SMORF proteins

#### 2.3.1 Peptide-specific antiserum

SMORF specific antiserum was prepared in rabbits using a peptide immunogen. A conserved peptide that was present in a predicted hydrophilic region of all SMORF ORFs could not be identified. Thus, a semi-conserved synthetic peptide 12 amino acids long (KYSVEWYLLPKP), which represented a total of 15 SMORF proteins, was synthesised (Biosynthesis, Inc., USA). The amino acid sequence of this peptide is strictly conserved in eight of the *smorf* encoded putative proteins (genes BBOV_I001170, BBOV_I005150, BBOV_II001380, BBOV_II001390, BBOV_III000050, BBOV_III002340, BBOV_III011960 and BBOV_IV007960). A single conservative amino acid change occurs in seven others (BBOV_III000690, BBOV_I003880, BBOV_II006800, BBOV_II000400, BBOV_II004150, BBOV_I003850 and BBOV_I001160), and the terminal nine amino acids are present in an additional nine sequences. Rabbits were immunised s.c. with 200 μg of peptide coupled to Keyhole Limpet Haemocyanin (KLH, Imject Maleimide-Activated mcKLH Kit, Pierce), initially using FCA, followed by three booster immunisations using incomplete Freund’s adjuvant (IFA, Biosynthesis Inc.). The rabbit immunisation protocol at Biosynthesis, Inc. was approved through both the National Institutes of Health Animal Welfare Assurance Program (#A3669-01) and the United States Department of Agriculture (research licence #23-R-0089).

#### 2.3.2 Recombinant SMORF protein

**Fig. 1:**
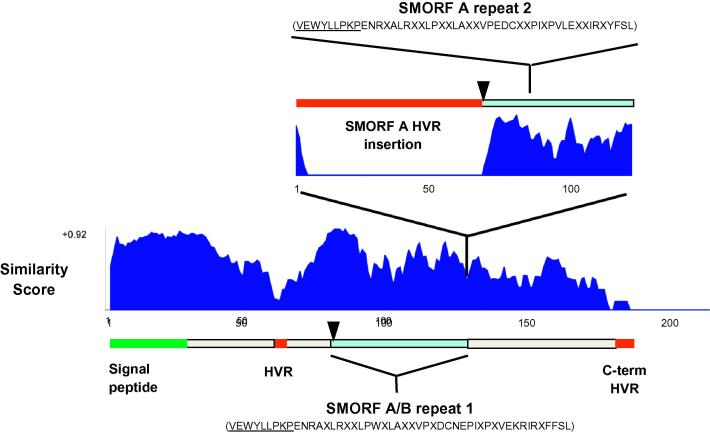
General topology of the small open reading frame (SMORF) family. The similarly score plot of SMORF proteins generated using the AlignX module of Vector NTI is presented. (A more detailed presentation of the analysis used to generate this figure is available in the Supplementary Fig. S1.) The signal peptide is designated in green and hypervariable regions (HVR) in red. SMORF A and B polypeptides differ by the presence of a large hypervariable insertion, with a similarity plot of the insertion shown above the main sequence. Degenerate repeat sequence regions are shown in light blue, with repeat 1 present in all SMORF polypeptides and repeat 2 present only in the SMORF A insertion. The location of the 12-mer peptide KYSVEWYLLPKP used for immunisation is identified with a black arrowhead and the C-terminal 9 amino acid portion of the peptide present in the repeat regions is underlined.

The complete BBOV_III007740 sequence, a *smorf* B gene (see Section 3.1) was selected for cloning and expression. This gene was selected since *smorf* A proteins could potentially induce antibodies against a variable sequence present only in that set of proteins (see Fig. 1), and because it had previously been demonstrated to be transcribed (Brayton et al., 2007). The gene was cloned into a pBAD/Thio-TOPO expression vector (Invitrogen) and transformed into *Escherichia coli*. After induction with arabinose, the *E. coli* extract was sonicated twice for 1 min each at 400 V in the presence of EDTA-free protease inhibitor (Roche), and was separated on a 12% SDS–PAGE gel followed by excision of the band representing the recombinant protein (37 kDa). The excised gel was dialysed (Spectrum, MWCO 12–14 kDa) and electroeluted overnight at 40 V. Purity of the electroeluted recombinant protein was confirmed in a second SDS–PAGE gel stained with Coomassie Blue.

#### 2.3.3 Protein isolation and immunoblots

Total protein was obtained from in vitro cultured Mo7.2 strain infected erythrocyte (22% parasitised erythrocytes) supernatants or T2Bo cryopreserved stabilates (∼1% parasitised erythrocytes) by washing erythrocytes three times with cold PBS with centrifugation between washes at 19,000*g* for 10 min at 4 °C, resuspending in PBS and freezing at −80 °C for 2 h to disrupt infected cells and merozoites. After thawing on ice, the disrupted parasites were washed three times as previously described and the pellet was resuspended in lysis buffer (0.1 M Tris, 10 mM EDTA, 2% NP-40) in the presence of EDTA-free protease inhibitor (Roche) and sonicated. Whole protein extract was stored at −80 °C until used. A negative control protein extract was obtained from uninfected erythrocytes using the same protocol.

Total protein extract (10 μg) was separated by 4–12% SDS–PAGE (Biorad Inc.) and transferred to a polyvinyl difluoride (PVDF) membrane (Amersham hybond-P, 45 μm). The PVDF membrane was blocked using I-Block (containing 0.2% casein in 0.1% Tween-20/PBS – Tropix, Applied Biosystem, USA) at 37 °C with shaking (200 rpm) for 2 h, followed by incubation with rabbit anti-SMORF peptide serum at a dilution of 1:750 overnight at 4 °C. After washing, bound antibodies were detected using goat anti-rabbit IgG coupled to alkaline phosphatase (1:10,000, Tropix, Applied Biosystem). Immunoblots were developed using CDP-Star Alkaline Phosphatase chemiluminescent substrates (Tropix, Applied Biosystem).

## 3 Results

### 3.1 Smorf gene family topology

To gain further insight into the *smorf* gene repertoire in the T2Bo strain that was used for genome sequencing, we first analysed and compared the sequences of the 44 T2Bo *smorf* gene family members identified in genome sequencing (Brayton et al., 2007). As previously reported, all paralogs except BBOV_I003850 have a signal peptide ranging from 19 to 27 amino acids in length as predicted by Signal P v. 3.0 (Krogh et al., 2001; Bendtsen et al., 2004; Brayton et al., 2007). BBOV_I001180 contains an insertion of 22 amino acids before the predicted signal peptide. It is currently unknown whether this insertion affects the processing or translocation of the encoded polypeptide. However, data generated in this study (see Section 3.3) indicates that BBOV_I001180 is transcribed. Following the signal peptide, the encoded polypeptides range in diversity from 95% identity between the most closely related pair of SMORF proteins to 25% identity between the most distantly related pair (AlignX, Vector NTI). A short hypervariable (HVR) block (see Fig. 1, designated in red) is present in the N-terminal half of all SMORF proteins. A similarly polymorphic C-terminal tail ranging from seven to 47 amino acids (Fig. 1) is present in 31/44 SMORFs.

The *smorf* genes can be subdivided into two groups, designated A (20 genes) and B (24 genes). *Smorf* A encoded polypeptides are interrupted by a large HVR insertion, ranging from 53 to 199 amino acids in length and predicted to encode a region with hydrophilic secondary structure (Fig. 1). A degenerate repeat (designated repeat 1) with the sequence VEWYLLPKPENRAXLRXXLPWXLAXXVPXDCNEPIXPXVEKRIRXFFSL is present in the central region of both SMORF A and SMORF B polypeptides (Fig. 1, light blue region). A similar sequence (designated repeat 2, Fig. 1 – VEWYLLPKPENRXALRXXLPXXLAXXVPEDCXXPIXPVLEXXIRXYFSL) is repeated within the SMORF A insertion (Fig. 1). The variable number of degenerate repeats in SMORF family members is responsible in large part for their variation in size.

Of the 44 *smorfs*, two (BBOV_II004150 and BBOV_IV006390) contain predicted introns. BBOV_II004150 has two putative introns; the first is 36 bp long and is located 78 bp downstream from the first adenine, while the second is 22 bp long and is located 432 bp downstream from the same nucleotide. BBOV_IV006390 has one putative intron that, at 968 bp in length, is unusually large. It is located 318 bp downstream from the first adenine. Confirmation of these sequences as introns has not been possible since transcripts from the genes containing them have not yet been identified (see Section 3.3).

### 3.2 Smorf gene strain differences

Sequence variability among strains has been reported for the large multigene families in Apicomplexan parasites (Blythe et al., 2009). To determine whether strain diversity was present in the *smorf* gene family, we designed specific primers to uniquely target PCR amplification of individual *smorf* genes (Supplementary Table S1). All primer sets were able to amplify a fragment of the predicted size for each of the 44 genes in T2Bo (two primer pairs were able to amplify two T2Bo genes). Amplicon identity was confirmed through sequencing.

**Fig. 2:**
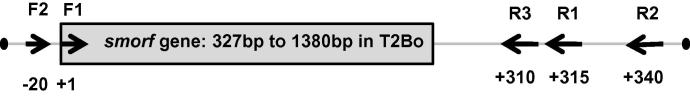
Location of degenerate primers for amplifying new *Babesia bovis* Mo7 strain *smorf* genes. The gray line represents the genome backbone. Black arrows show the positions of the primers. A generic *smorf* gene is represented by the light gray box. Primer F1 is located at the 5′ end of the open reading frame. Sequences of the primers are indicated in Section 2.2.4. Numbers indicate the predicted amino acid number.

**Fig. 3:**
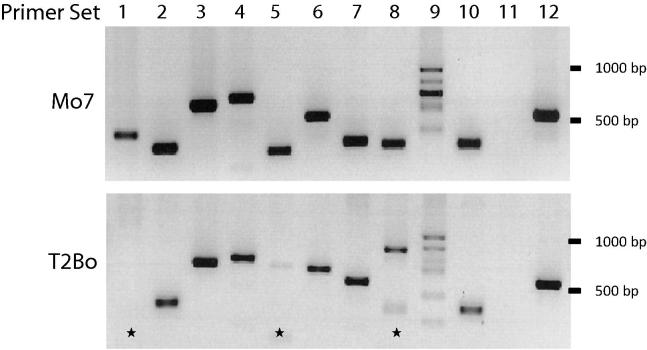
Amplification of *smorf* genes using 12 different *Babesia bovis* Mo7 strain-specific primers. Primers used are designated by numbers across the top. (See Supplementary Table S2 for primer sequences.) The stars mark lanes with either no amplicon or a size difference in the amplicons between the two *B. bovis* strains. Gradient PCR was performed to determine optimal conditions for primer set 9 (data not shown), but it consistently amplified multiple genes. Primer set 11 did not amplify a product in either strain.

The T2Bo strain was originally isolated from southern Texas near the Texas – Mexico border. To determine whether the same set of *smorf* genes was shared in a geographically related strain, the same set of primers was used to amplify gDNA from the Mo7 strain, originally derived from an isolate obtained in a *B. bovis* endemic region of northern Mexico, relatively near to where the T2Bo strain was found. Only 22 of the 44 predicted *smorfs* (all of which were identical in sequence to the T2Bo gene) were amplified in the Mo7 strain of *B. bovis*, suggesting that the primer sequence, and likely the *smorf* gene repertoire, varied in the Mo7 strain. (One primer set identified an Mo7 gene which differed in sequence from the T2Bo counterpart for which the primers were designed – see below.) To determine whether these *smorf* genes were entirely missing or whether there was sequence divergence between strains, two forward and three reverse degenerate primers were designed to target nucleotides in the 5′ and 3′ flanking regions of the T2Bo *smorf* genes that were not able to be amplified in Mo7 (primers F2 and R1–3, Fig. 2) or the initial 5′ sequence of the *smorf* ORF (Primer F1, Fig. 2). After sequencing 35 clones from each amplification reaction using combinations of these degenerate primers (a total of 210 clones), 12 additional *smorf* genes were identified in Mo7 that differed in sequence from known *smorf* genes in T2Bo. Specific primers (Supplementary Table S2) were subsequently designed for each of these “novel” Mo7 *smorf* genes to confirm their presence in Mo7 and to investigate potential paralogs previously unknown in T2Bo. Eleven of 12 primer pairs successfully amplified a band of the expected size in Mo7 (Fig. 3). Primers for seven of these 11 Mo7 *smorfs* were also able to amplify a band of similar size and identical sequence in T2Bo, while two other primer sets amplified bands of unexpected size or multiple bands (Fig. 3). Despite optimisation of amplification conditions, primer set 9 (9F and 9R) amplified multiple DNA fragments in both strains. However, an amplicon corresponding to the predicted size for primer set 9 was sequenced in both Mo7 and T2Bo and was identical to the expected sequence. Remaining bands amplified with this primer set were not sequenced. Primer set 11 was never able to amplify a fragment in Mo7 or T2Bo, suggesting that the gene identified using degenerate primers could have been an in vitro artefact from recombination.

In total, using degenerate and specific T2Bo primers, 12 Mo7 *smorf* genes not previously seen in the T2Bo genome sequence were identified (Table 1). These include 11 genes identified using degenerate primers and one Mo7 gene identified using T2Bo gene-specific primers but with a sequence different from the T2Bo counterpart from which primers were designed. To determine which *smorf* genes these newly identified Mo7 genes were most closely related to, we compared their sequences to the entire *smorf* gene repertoire in T2Bo and Mo7. Two of the 12 newly identified Mo7 SMORF proteins (Mo7 sequence Nos. 1 and 7 – Table 1) have the greatest percentage identity (ID) with *smorf* encoded proteins common to these two strains (“core repertoire”). Three newly identified SMORF proteins (Mo7 sequence Nos. 2, 4, and 8 – Table 1) have a greater sequence ID to non-core T2Bo SMORF proteins than any other “novel” Mo7 SMORF, while seven of the new Mo7 proteins (3, 5, 6, 9–12) have a greater percentage ID with other “novel” Mo7 *smorf* encoded proteins than with any known T2Bo SMORF or T2Bo/Mo7 shared core SMORF protein (Table 1). When T2Bo non-core proteins are compared in the same manner, three are most closely related to the common set of proteins, 11 most closely match others found only in the T2Bo repertoire and six most closely match novel Mo7 *smorf* encoded proteins. Collectively, these results suggest that some *smorf* genes in the species repertoire are undergoing lineage-speciation in a strain dependent manner.

### 3.3 Transcriptional profile of smorf genes

It was previously reported that multiple *smorf* genes are transcribed in the uncloned T2Bo strain used for genome sequencing (Brayton et al., 2007). The previous analysis used degenerate primers to amplify cDNA and the amplified *smorf* transcript sequences were different from the *smorf* repertoire predicted from genome sequencing (Brayton et al., 2007). While there are several possible explanations of this result, the use of degenerate primers could have resulted in in vitro recombination. To thoroughly examine specific *smorf* gene transcription and attempt to avoid this possibility, we used specific primer sets designed for each of the 44 *smorf* genes (Supplementary Table S1) to amplify cDNA generated by random decamers from *B. bovis* transcripts. All but three primer pairs were able to specifically amplify the expected *smorf* gene, while 3/44 amplified more than one gene. For primers that amplified more than one gene, restriction enzyme sites that could differentiate the amplicons were identified and used in the transcriptional analysis to specifically identify the gene being transcribed.

We initially examined *smorf* transcription using total RNA isolated from the cerebral cortex of a calf experimentally infected with T2Bo strain parasitised erythrocytes. Brain tissue was collected following death of this animal from acute clinical babesiosis with signs referable to anaemia and neurological disease (see Section 2.2.1). Selective accumulation of parasitised cells suggestive of sequestration was confirmed by histological examination (data not shown). The percentage of parasitised erythrocytes within brain capillaries throughout the brain was ⩾90%, while in skin it was ∼50%, in liver and spleen ∼1%, and in peripheral blood on the day of death <0.05%. Transcripts from 32 of 44 *smorf* genes were amplified from this population of parasites. Among the 21 unpaired *smorf* genes in the T2Bo genome, 15 were transcriptionally active. All *smorf* genes that occur as pairs in either a head to tail or head to head orientation, or are clustered as more than one pair in the genome (Brayton et al., 2007) were transcriptionally active. Both genes were transcribed in five pairs, while only one of the two paired genes was transcribed in five other pairs. In the single *smorf* gene locus containing three tandemly arranged genes, the two external genes in the locus were transcribed, while the middle gene was transcriptionally silent in this analysis. Collectively the data indicate that neither clustering nor pairing of *smorf* genes is required for gene expression, as previously reported for *B. bovis ves1* genes (Al-Khedery and Allred, 2006).

The large number of *smorf* transcripts that were detected in brain-sequestered T2Bo parasites was unexpected as dominant expression of a single gene from a multigene family is more common, particularly in a specific tissue (Duffy et al., 2005). This observation could be due to a mixed parasite population in the infected calf. To determine whether a clonally derived line of *B. bovis* would transcribe a more limited repertoire of *smorf* genes, we examined in vitro derived RNA from a recently cloned Mo7 laboratory strain (termed Mo7.2) for the presence of *smorf* gene transcripts. The newly derived clone Mo7.2 was obtained by limiting dilution for this analysis since the original Mo7 strain has been cultured for many years following original cloning. Since the *smorf* repertoire in this strain is different from T2Bo, only the common “core” of 22 genes was examined. Results demonstrated that 19/22 core genes were transcriptionally active in Mo7.2. Two of the three transcriptionally silent genes in Mo7.2 (BBOV_III000020 and BBOV_III002350) were transcribed in T2Bo. Transcriptionally active Mo7.2 genes included those with homologues in T2Bo that are both paired and unpaired.

### 3.4 SMORF protein expression

**Fig. 4:**
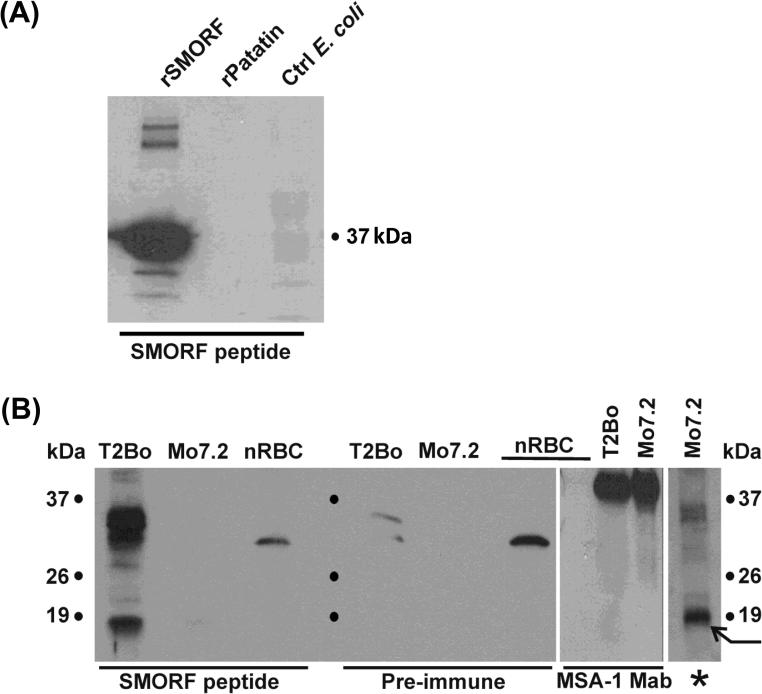
Immunoblot showing SMORF expression in *Babesia bovis* T2Bo and Mo7.2 strains. (A) Electroeluted recombinant SMORF (rSMORF), control recombinant patatin from *Anaplasma marginale* (rPatatin), and control non-recombinant *Escherichia coli* extract (Ctrl *E. coli*) were electrophoresed and immunoblotted with anti-SMORF peptide serum. (B) Antigen preparations (from *B. bovis* strain, parasites and control) as indicated along the top of the panel were electrophoresed and immunoblotted with anti-SMORF peptide or pre-immune rabbit serum as indicated below the panel. nRBC refers to an antigen preparation of uninfected bovine erythrocytes. The panel immunoblotted with monoclonal antibody raised against *B. bovis* merozoite surface antigen-1 (MSA-1 Mab) shows that the T2Bo and Mo7.2 lanes are loaded with equal amounts of parasite protein. The lane marked with an asterisk is an overexposure of the Mo7.2 lane immunoblotted with SMORF peptide antiserum, and the arrow points to the prominent 18 kDa polypeptide. Multiple bands are present in T2Bo, but in Mo7.2 are only seen when the immunoblot is overexposed.

Having identified a large number of transcribed *smorf* genes in both culture- and tissue-derived *B. bovis*, and in both uncloned and cloned parasite populations, we next examined whether these transcripts were translated using SMORF peptide-specific antiserum. Due to the extensive sequence variation in the *smorf* gene family, only two overlapping hydrophilic peptides could be identified that were strictly conserved in a large number of SMORF sequences but not present in any other predicted protein sequence in the *B. bovis* genome. Antibodies were generated in rabbits against one of these peptides (KYSVEWYLLPKP) which was represented in 15 SMORF proteins with complete identity or a single conservative amino acid change. An immunoblot using electroeluted recombinant SMORF protein and multiple controls including an unrelated, recombinant protein (rPatatin from *Anaplasma marginale*) and *E. coli* lysate, demonstrated that antibodies had been generated that would strongly bind SMORF polypeptides (Fig. 4A). The peptide-specific antiserum also bound in immunoblots to multiple polypeptides of a size range predicted from *smorf* genes of both the uncloned T2Bo and the cloned Mo7.2 parasites (Fig. 4B). There were differences between the two strains in the number and intensity of polypeptides recognised by the antiserum. In T2Bo, three polypeptide bands, two at ∼31–33 kDa and one at ∼18 kDa were the most prominent, while less intense bands were present at ∼21, 27, 43 and 44 kDa. In Mo7.2, the most strongly recognised polypeptide was at ∼18 kDa (Fig. 4, arrow) and there appeared to be fewer polypeptides detectable in Mo7.2 than in T2Bo. Since an equivalent number of parasitised cells and quantity of protein from each strain were loaded in each lane (as confirmed by the similar staining intensity of positive control protein *B. bovis* merozoite surface antigen-1), these differences suggest strain variation in the gene products that are expressed and the amount of individual SMORF protein present. A ∼30 kDa protein present in both normal erythrocyte controls and parasite lanes is recognised by both control pre-immune and immune sera, and was therefore not identified as a SMORF polypeptide.

## 4 Discussion

The Apicomplexan intraerythrocytic parasites *Plasmodium*, *Theileria* and *Babesia* are significant causes of human and animal morbidity and mortality, particularly in lower income countries (Bishop et al., 2004; Bock et al., 2004; Lau, 2009; Kappe et al., 2010). After an acute phase of clinical disease, all three hemoparasites have evolved mechanisms which enable avoidance of an immune response to clear infection, thus allowing their persistence in their respective mammalian hosts. Multigene families play a crucial role in facilitating persistence and mediate functions such as invasion of the host cell, antigenic variation and cytoadhesion (Morrison, 2007; Dzikowski and Deitsch, 2009).

PfEMP1 encoded by the *var* multigene family, has been extensively investigated and its role in immune evasion and persistence is well described (Pasternak and Dzikowski, 2009). The PfEMP1 functional homologue, VESA1, is encoded by the *ves1* multigene family in *B. bovis*, and plays a similar pivotal role in babesial pathogenesis (Allred and Al-Khedery, 2004). Also described are a series of large gene families that are most often closely associated in the genome with *var*, *ves1* or their homologues in other species. These include the *rifin* and *stevor* genes in *P. falciparum* and *yir* in the rodent malaria species *Plasmodium yoelii yoelii* (Cunningham et al., 2005). A similar multigene family closely associated with *ves1* genes was also discovered in *B. bovis* during genome sequencing (Brayton et al., 2007) and was termed “*smorf*” for “small open reading frame”. While most multigene families cluster in subtelomeric locations, the *ves1* and *smorf* genes in *B. bovis* are distributed across all four chromosomes (Brayton et al., 2007).

Phylogenetic analysis in Apicomplexans has revealed a high degree of interspecies divergence for these multigene families, indicating a rapid rate of mutation – a trend common among species-specific gene families (Kuo and Kissinger, 2008). Variation among strains has also been described (Blythe et al., 2009) and the *smorf* gene family is no exception. In this study, we identified a core set of 22 *smorf* genes shared between the T2Bo and Mo7 *B. bovis* strains, both of which were originally isolated in a geographically contiguous area of southern Texas and northern Mexico. Interestingly, however, specific primers for 22 of the other *smorf* genes present in the T2Bo strain would not amplify a sequence in the Mo7 strain. It was possible that the original uncloned T2Bo contained multiple parasite populations, each of which had a different, but more limited, gene repertoire, while the clonal Mo7 strain consisted of a single population with fewer *smorf* genes. However, using combinations of degenerate primers targeting flanking regions or the initial 5′ sequence of the *smorf* ORF, followed by sequencing 210 of the resulting clones, we were able to identify 11 new *smorf* gene sequences that could be verified by subsequent gene-specific PCR and sequencing. As shown in Table 1, these novel Mo7 *smorf* genes, together with one identified using T2Bo gene-specific primers, were all related to genes in the T2Bo *smorf* repertoire and in some cases had greater identity to known T2Bo genes than to other newly identified Mo7 genes. The same is true of T2Bo *smorf* genes not in the common set. However, in both strains some *smorf* genes are most closely related to another *smorf* sequence in the same strain, indicating lineage-specific evolution of these genes. Strain and isolate variation has been well characterised for merozoite surface proteins in the variable merozoite surface antigen (VMSA) family (Berens et al., 2005; LeRoith et al., 2006; Lau et al., 2010), which includes only 3–4 gene copies per genome (Florin-Christensen et al., 2002). Much less is known about *ves1* strain variation in *B. bovis*. However, as in related hemoparasites, our results suggest that there is selective pressure for continuous evolution of these larger multigene families (Albrecht et al., 2006).

Transcription of *smorf* genes was initially examined in parasites obtained from T2Bo-infected bovine brain. The animal from which tissues were obtained died from acute clinical babesiosis typical of the T2Bo strain (Bastos et al., 2010). There was selective, disproportionate accumulation of parasitised cells in the brain of this animal compared with other tissues and peripheral blood. There is no clear case definition for sequestration in vivo and we cannot rule out that some of the infected erythrocytes in the brain have accumulated for other reasons, for example local coagulatory abnormalities. However, the clinical signs were consistent with neurobabesiosis and histological findings were suggestive of sequestration. Thus, we hypothesised that this location would potentially contain a selected subpopulation of parasites expressing genes that enable parasitised erythrocytes to adhere to brain endothelial cells. If consistent with expression and apparent selection of *ves1* genes, *smorf* gene transcription might be more limited than in unselected parasites. Surprisingly, 32/44 different *smorf* gene transcripts were identified. Unlike the finding in the original description of *smorf* genes (Brayton et al., 2007), all 32 transcripts had an identical sequence to a *smorf* gene reported in the genome. While it is possible that the original generation of transcripts resulted in some arising from in vitro recombination, the genome contains a large gap which could also contain the genes corresponding to these “orphan” *smorf* transcripts. It would have been less likely in this study to identify transcripts from *smorf* genes potentially present in the gap using gene-specific primers, and it is possible that the entire repertoire of *smorf* transcription is larger than found if potential genes in the gap are also transcriptionally active. Transcription occurred from both paired and unpaired *smorf* genes, unlike the mutually exclusive transcription that has been identified in the *ves1alpha* gene family of *B. bovis* (Zupanska et al., 2009).

Since the T2Bo strain is an oligoclonal population (Perez-Llaneza et al., 2010), we analysed transcription from a clonal line of *B. bovis* grown in vitro. The Mo7 strain clone was originally derived in 1983 (Rodriguez et al., 1983) and has been passaged continuously in vitro. For the purpose of this study, we re-cloned this line of Mo7 through two rounds of limiting dilution. Nineteen of the core 22 *smorf* genes were found to be transcriptionally active in this recently cloned line. Again, messages were identified from genes whose homologues in T2Bo are in both paired and unpaired arrangements.

The PCR procedure used in these experiments included 40 cycles of amplification. Our purpose was not to quantitate transcript levels from different genes, only to show that they were expressed. So while we may have identified “rare” transcripts using this sensitive procedure, it is also possible that “promiscuous” transcription is a feature of *B. bovis smorf* gene expression. It is also possible that individual parasites in the population have a dominant *smorf* gene transcribed, while in a population that is not under selection from immune or other selective forces, the transcriptional profile will consist of a mix of *smorf* messages, including in clonally derived lines. This pattern of transcription is similar to transcription of the *yir* multigene family in *P. yoelii* (Cunningham et al., 2005, 2009).

The *smorf* genes were initially identified through genome sequencing and were analysed for transcription but not protein expression. To determine whether one or more protein products were produced from the multiple transcripts identified through RT-PCR, we generated antiserum against a peptide strictly conserved or with only one conservative amino acid change in 15 of the T2Bo *smorf* encoded proteins. Immunoblots of T2Bo strain *B. bovis* proteins from cryopreserved, peripheral blood-derived parasites showed multiple polypeptides of varying molecular mass consistent in general with the size predicted from transcripts and with at least three more prominent than the others. The 12-mer peptide selected for immunisation shares continuous six amino acid ID with several host proteins and five amino acid ID with six *B. bovis* proteins. All of the prominent polypeptides bound by anti-peptide antibodies were of a lower molecular weight than predicted for other *B. bovis* proteins sharing a stretch of five amino acids. Pre-immune serum bound strongly to a ∼30 kDa host polypeptide in normal erythrocytes, so the *smorf* specificity of a similar sized polypeptide in infected erythrocytes cannot be determined. The recently cloned Mo7.2 strain was also examined to determine whether a recently cloned parasite line would express multiple or a single dominant SMORF protein. The peptide sequence is present with 100% ID in eight Mo7.2 SMORF proteins (six core and two Mo7.2 specific), and with a single conservative substitution in four more. Although multiple polypeptides were again recognised by SMORF peptide-specific antibodies in the clonal Mo7.2 strain, only one dominant protein band was present at 18 kDa (arrow in Fig. 4B). The presence of at least three prominent proteins in the uncloned T2Bo strain and only one strong band in the Mo7.2 clonal line could reflect the oligoclonal population of parasites in T2Bo with one dominant population in Mo7.2. However, our results must be interpreted as preliminary only since our goal was to confirm expression rather than to comprehensively catalogue all SMORF proteins expressed. It is unknown how many *smorf* gene products the peptide-specific antibodies are capable of binding. The peptide selected to generate antiserum was strictly conserved or had a single conservative substitution in a limited (15 in T2Bo) number of predicted SMORF proteins. There is only minor amino acid variation in the peptide sequence or surrounding sequence in a number of other SMORF proteins, and the C-terminal nine amino acids of this peptide are present in 24 total SMORF proteins. Thus, less prominent bands in either strain could be a result of less well represented parasite subpopulations expressing these SMORF proteins, SMORF proteins with epitopes not as well recognised by the anti-peptide serum, varying production or trafficking of SMORF proteins encoded by different *smorf* genes in a single parasite, or breakdown products of larger SMORF proteins.

Multigene families in related hemoparasites typically traffic to the erythrocyte cytoplasm and membrane, with variable surface exposure. We attempted to determine whether the SMORF proteins were transported and located similarly in infected erythrocytes using immunolocalisation. However, due to discordant results using different reagents, we were unable to demonstrate conclusively where SMORF proteins localised within the merozoite or whether they were present at the infected erythrocyte membrane. Further studies are necessary to resolve this question, as well as to determine the function of these novel proteins.

In summary, we have demonstrated that proteins encoded by the novel *smorf* multigene family in *B. bovis* are expressed, that the repertoire of *smorf* genes is larger than previously reported, and that there is significant variation and differential amplification of this family within different strains. In contrast to what has been shown with transcription of the *ves1* gene family from a single locus of active transcription, the data demonstrate transcription from multiple *smorf* gene loci in a clonal line, indicating that while spatially related at the genome level, the biology of these two gene families may differ when compared at the transcriptional level. However, we have not determined whether only a single locus of transcription is active in a single infected cell or parasite. In addition, multiple polypeptides are translated. The possibility remains, however, that there is control or selection at the population level for translation of only one or a few *smorf* gene products. Future studies will address remaining important questions about the *smorf* gene family, including the cellular localisation and function of SMORF proteins in both mammalian and tick hosts, their immunogenicity and their role, if any, in antigenic variation and persistence.

## Figures and Tables

**Table 1:** Similarity of newly identified Mo7 SMORF sequences to previously identified T2Bo SMORF proteins.

New Mo7 Seq No.	Deduced amino acid sequence with highest similarity			
	In T2Bo		In Mo7	
	Gene name^a^	% ID	Gene name	% ID
1	**BBOV_I001120**	71	**BBOV_I001120**	71
2	BBOV_III002340	94	6	85
3	**BBOV_I001420**	50	11	57
4	BBOV_III000690	63	6 and 7	51
5	BBOV_III002340	61	3	85
6	**BBOV_I001130**	58	13	72
7	**BBOV_III002350**	70	**BBOV_III002350**	70
8	BBOV_I001180	83	12	66
9	**BBOV_II006800**	53	11	72
10	BBOV_III002340	62	10	72
11	**BBOV_II006800**	54	9	66
12	**BBOV_II004220**	63	7	72

ID, identity.

a Genes highlighted in bold represent *smorf* sequences that are common to both T2Bo and Mo7 and comprise part of the “core repertoire”.

## Appendix A Supplementary data

Supplementary Fig. S1 Background data for analysis and generation of Fig. 1. (A) The alignment of the 44 SMORF polypeptides. Insertions that occur in only one or two sequences have been removed and these sequences are indicated by the designation “minus ins”. Yellow background indicates absolutely conserved residues, blue background are residues conserved in the majority of sequences in the alignment, while green background indicates conserved residues. Numbers in the parenthesis indicate amino acid number. Similarly score plots of SMORF proteins generated using the AlignX module of Vector NTI are presented in B, C and D. The alignment shown in A results in the similarity profile depicted in B. (C) Demonstration of the similarity plot when the SMORF B proteins are removed. (D) The similarity plot for the final Fig. 1.

Supplementary Table S1 Primer sequences for *Babesia bovis* T2Bo strain *smorf* genes.

Supplementary Table S2 Primer sequences for *Babesia bovis* Mo7 strain *smorf* genes.
