# Supplementary figures and images for: Expression and strain variation of the novel “small open reading frame” (smorf) multigene family in Babesia bovis
Source: Int J Parasitol. 2012 Feb;42(2):131–8. doi: 10.1016/j.ijpara.2011.10.004 (PMC3459096; doi:10.1016/j.ijpara.2011.10.004)

B.

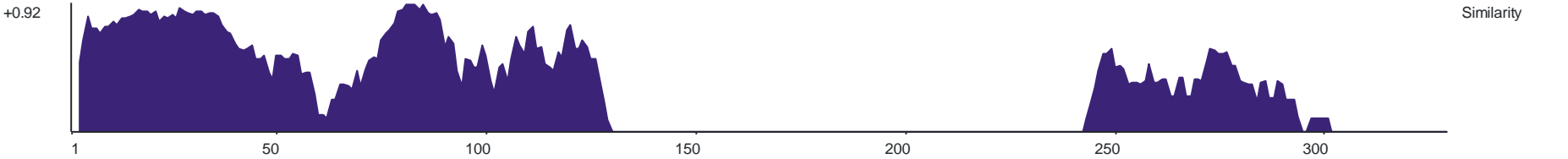

C.

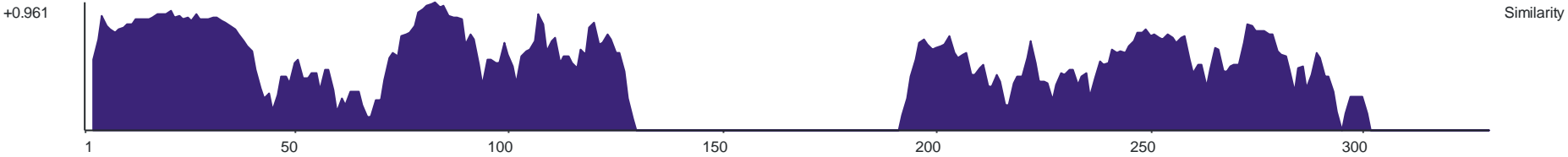

D.

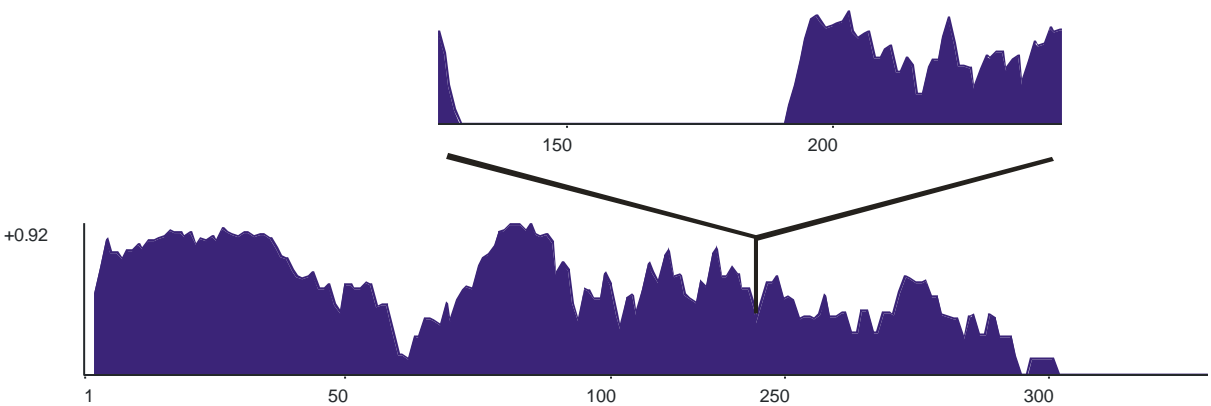

Supplement: Supplementary Fig. S1 — Background data for analysis and generation of Fig. 1. (A) The alignment of the 44 SMORF polypeptides. Insertions that occur in only one or two sequences have been removed and these sequences are indicated by the designation “minus ins”. Yellow background indicates absolutely conserved residues, blue background are residues conserved in the majority of sequences in the alignment, while green background indicates conserved residues. Numbers in the parenthesis indicate amino acid number. Similarly score plots of SMORF proteins generated using the AlignX module of Vector NTI are presented in B, C and D. The alignment shown in A results in the similarity profile depicted in B. (C) Demonstration of the similarity plot when the SMORF B proteins are removed. (D) The similarity plot for the final Fig. 1. [file mmc1.pdf]
