# Supplementary material for: Expression and strain variation of the novel “small open reading frame” (smorf) multigene family in Babesia bovis
Source: Int J Parasitol. 2012 Feb;42(2):131–8. doi: 10.1016/j.ijpara.2011.10.004 (PMC3459096; doi:10.1016/j.ijpara.2011.10.004)
Supplement: Supplementary Table S1 — Primer sequences for Babesia bovis T2Bo strain smorf genes. [file mmc2.doc]

**Supplementary Table S1**

Primer sequences for *Babesia bovis* T2Bo strain *smorf* genes.

| Gene | Primer: name and sequence | Amplicon length (bp) |
| --- | --- | --- |
| BBOV_I001120 | F 5’ CAGTTTCTCTAAGCTCTGTGTAGTTGTGGC 3’ | 700 |
|  | R 5’ GTTTTCCGGCTTGGGCAGATGATAC 3' |  |
| BBOV_I001130 | F 5’CTACCTTCCATTGCCACTTCTACCGATG 3’ | 358 |
|  | R 5’ GCGGCAAGGTTAACAGATACCAGTC 3’ |  |
| BBOV_I001160 | F 5’ CTCTGTTTAGTGGTGGCATTTGGGC 3’ | 479 |
|  | R 5’ CGTTTGACAACTTCAGGCTTTATGGGTTC 3’ |  |
| BBOV_I001170 | F 5’ GAAGCTCTGTGTAGTGGTGGCATTC 3’ | 443 |
|  | R 5’ CCGGAACCTTTGAGGCCAAATTAC 3’ |  |
| BBOV_I001180 | F 5’ ATGGTAGCCTTCAASATGTTATGGAAGC 3’ | 664 |
|  | R 5’ CCCTAATGCGTTTTTCCAGTACGG 3 |  |
| BBOV_I001370 | F 5’ ATGGTAGCCTTCAASATGTTATGGAAGC 3’ | 567 |
|  | R 5’ CGAATTGCCTTGATACGACCATCC 3’ |  |
| BBOV_I001420 | F 5’ GTAGCAGAACATGGCACGGAGTCTC 3’ | 751 |
|  | R 5’ GCATGTACCATGTAGCGGCATACAAC 3’ |  |
| BBOV_I003850 | F 5’ ATGGAATCTCTCTCTAGTTCTGGCATTCGTG 3’ | 310 |
|  | R 5’ CTATGGGCTCATCACAGTCCTTAAGAACG 3’ |  |
| BBOV_I003860 | F 5’ CTTTCTAGTTGTGGCTCTCGGGTTC 3’ | 277 |
|  | R 5’ CTCCTCTGGAACCATTGCAGCCAAATC 3’ |  |
| BBOV_I003880 | F 5’ CCAAGGATCTGTGTAGTTGTGGTATTTGGG 3’ | 586 |
|  | R 5’ CTGCTAGGTCAAATGGCAACTCGTTAC 3’ |  |
| BBOV_I003890 | F 5’ ATGGTAGCCTTCAASATGTTATGGAAGC 3’ | 664 |
|  | R 5’ CCCTAATGCGTTTTTCCAGTACGG 3' |  |
| BBOV_I005150 | F 5’ GTAGCCTTCAACATGCTATGGAAAGTCTGTCTG 3’ | 544 |
|  | R 5’ GCCTCTGCTGTTCCTCACTGTATG 3’ |  |
| BBOV_II000060 | F 5’ ATGGTAGCCTTCAASATGTTATGGAAGC 3’ | 613 |
|  | R 5’ CACCTTTGGCCAGATCGCTTGGTAAC 3’ |  |
| BBOV_II000400 | F 5’ CAACAGATTCCCCCGTAAGAATGAAGAAC 3’ | 346 |
|  | R 5’ CATACTCTCTGGTTTCGGTACCTCTTGAG 3’ |  |
| BBOV_II001380 | F 5’ CAAAGCTCTGTGTATTAGTGGCCTTCGG 3’ | 703 |
|  | R 5’ CCCCTAATACGTCTTTCTGTTGCTGGAGA 3’ |  |
| BBOV_II001390 | F 5’ CAAAGCTCTGTGTATTAGTGGCCTTCGG 3’ | 406 |
|  | R 5’ CGTAGAGCGGTTCTATTTTCCGGCT3’ |  |
| BBOV_II002280 | F 5’ CATCCCAAGTACGACGTAGAATGGTATCTG 3’ | 864 |
|  | R 5’ CCAATGGAAACCACATGACAATCGACTC 3’ |  |
| BBOV_II002290 | F 5’ CTCTAAGCTCTGTGTAGTGGTGGCATTGG 3’ | 341 |
|  | R 5’ CTCTAATACGCTCTTCCACCTTGGGATC 3’ |  |
| BBOV_II004150 | F 5’ GAGTGAGACTGATGAACCACCCAAG 3’ | 660 |
|  | 5’ CGATTGGGCCTTTGCTTCTTCTC 3’ |  |
| BBOV_II004160 | F 5’ CAGCTACCTCTACTGATGTAGCCCAAG 3’ | 246 |
|  | R 5’ CTCCTCTGGAACCATTGCAGCCAAATC 3’ |  |
| BBOV_II004220 | F 5’CTCTGTCACTGCTACTGATGTATCCCAAG 3’ | 398 |
|  | R 5’ GCTTCCTAATGTCGTTCTCCACTAAAGGTG 3’ |  |
| BBOV_II006800 | F 5’ CACTAAACGTCAAGAGGTACCTGAACC 3’ | 280 |
|  | R 5’ CCAACCGGTACAATATAATAGTGCACCCC 3’ |  |
| BBOV_II006810 | F 5’ GGCATTTATTACCCAAGCCGGAAAATAGAGC 3’ | 254 |
|  | R 5’ CCACATTCGCTTGTTGCAGTCCTCTG 3’ |  |
| BBOV_II007780 | F 5’ GCTATGCGCAGTTGTGGCATTCG 3’ | 389 |
|  | R 5’ CGTATGAAGCTAAACGCTTTGCCTGTTGC 3’ |  |
| BBOV_II007820 | F 5’ CCACATGATTTGCTCGATGCTCTGCCAG 3’ | 438 |
|  | R 5’ GGAGTCGTTATTTAGCTGCGGGAAATCTTG 3’ |  |
| BBOV_III000020 | F 5’ CTCTGTGTGGTTGTGGCATTCGGG 3’ | 411 |
|  | R 5’ GGAGTCGTTATTTAGCTGCGGGAAATCTTG 3’ |  |
| BBOV_III000050 | F 5’ ATGGTAGCCTTCAASATGTTATGGAAGC 3’ | 702 |
|  | R 5’ TCACCTCAAACCTGGCATAGT 3’ |  |
| BBOV_III000690 | F 5’ GTTCTCTGCCACGGCTACCTCTAC 3’ | 372 |
|  | R 5’ GTGAAAGTATCTGAATGTAGACGCCTTGAC 3’ |  |
| BBOV_III001320 | F 5’ GTGCAGTGGTGGCATTTGGACTTTC 3’ | 348 |
|  | R 5’CTAGTATCTCCAACAGCCACCTAATGTC 3’ |  |
| BBOV_III002340 | F 5’ ATGGTAGCCTTTAACACTTTCTCCAAGC 3’ | 669 |
|  | R 5’ CTCTGGAACCGTTTCTGCCACG 3’ |  |
| BBOV_III002350 | F 5’ CTTTCTCCAAGCTCTGTGTAGTTGTCG 3’ | 336 |
|  | R 5’ GTTCTTCCAGCTCAGAGTCTAGTGGCTTG 3’ |  |
| BBOV_III007710 | F 5' ATGGTAGCTCTTAACATGTTATGG 3' | 327 |
|  | R 5' CTATTGCCTCATTACAGTCATATG 3' |  |
| BBOV_III007740 | F 5' ATGGTAGCTCTTAACATGTTATGG 3' | 561 |
|  | R 5' TCAAGATGGACGCATTGCC 3' |  |
| BBOV_III011930 | F 5’ GATTCTTTTGTGAGAATGGCGACATTGCCAC 3’ | 607 |
|  | R 5’ GCCCTATTTTCCGGCAAAGGTAACAGATACC 3’ |  |
| BBOV_III011960 | F 5’ GAAGGATTGGACAAAGGAGTCTGCAGCAAG 3’ | 444 |
|  | R 5’ CCTCTGCATTCTCTCACTGTAGGAG 3’ |  |
| BBOV_IV000040 | F 5’ CTAAGCTCTGTCTCGTGGTGGCATTTG 3’ | 314 |
|  | R 5’ CCACATTCGCTTGTTGCAGTCCTCTG 3’ |  |
| BBOV_IV000090 | 5' ATGGTAGCCTCAACACATTAC 3' | 423 |
|  | R 5' TTAGAAACCATATGAAGCTAAACGC 3' |  |
| BBOV_IV006390 | F 5' ATGGTAAATGTTAACAGATTACCAAAG 3' | 1380 |
|  | R 5' TTACAAATGCAAATACCAAAAACG 3' |  |
| BBOV_IV006420 | F 5’ ATGGTAGCCTTCAASATGTTATGGAAGC 3’ | 1176 |
|  | R 5’ AATCCCGTGGAAATATGAATGTAGACGC 3’ |  |
| BBOV_IV006430 | F 5’ GGCTCTCTGACACTGTCACTGCTACTG 3’ | 197 |
|  | R 5’ CGGCAACTCTTCACGAAGTTCTTTTCTGC 3’ |  |
| BBOV_IV007970 | F 5’ CCATGGACCATGATATTGTCCGTACCAGAGG 3’ | 499 |
|  | R 5’ GTTCATTATAGTCCTCTGGAACCTTGGCAG 3’ |  |
| BBOV_IV007960 | F 5’ CAAGCAGCCATAACACCCAAGGATACAGTG 3’ | 268 |
|  | R 5’ GGCAACACATAACGTAATGCGGCTC 3’ |  |
| BBOV_IV007930 | F 5’ CTCTCTGCCACTGTCACCTCTAC 3’ | 556 |
|  | R 5' CGCCTAATTCGCTTGGTAATTCCTCACGAAG 3’ |  |
| BBOV_IV012140 | F 5’ CTAAGCTCTGTCTCGTGGTGGCATTTG 3’ | 314 |
|  | R 5’ CCACATTCGCTTGTTGCAGTCCTCTG 3’ |  |
