# Supplementary material for: Expression and strain variation of the novel “small open reading frame” (smorf) multigene family in Babesia bovis
Source: Int J Parasitol. 2012 Feb;42(2):131–8. doi: 10.1016/j.ijpara.2011.10.004 (PMC3459096; doi:10.1016/j.ijpara.2011.10.004)
Supplement: Supplementary Table S2 — Primer sequences for Babesia bovis Mo7 strain smorf genes. [file mmc3.doc]

**Supplementary Table S2**

Primer sequences for *Babesia bovis* Mo7 strain *smorf* genes.

| Primer designation | Primer sequence | Amplicon length (bp) |
| --- | --- | --- |
| **1F** | 5’ GGAAGCTCTGTGCAATTGGGC 3’ | 409 |
| **1R** | 5’GGCTTGCGACAGTTCACTGG 3’ |  |
| **3F** | 5’ GGGTGAAGAATCGGCTACGAAACTTCACAAG 3’ | 633 |
| **3R** | 5’ GAAGGTAGAAGCATTGACTTCTGCTTTTGC 3’ |  |
| **4F** | 5’ CCCAAGGCGGAAAACAGAGC 3’ | 690 |
| **4R** | 5’ CAAGACATAACGTAAGGCATTTCTACTTTCCG 3’ |  |
| **5F** | 5’ GGTAAGAGAGAAGAACCTGCAACGAAGC 3’ | 283 |
| **5R** | 5’ GAGGGCAGATAATATATGAATACCGACGCC 3’ |  |
| **6F** | 5’ ATGTTCACCGTTGAATGGTATCTCTTACCC 3’ | 510 |
| **6R** | 5’ CCTCCTAATGTCTTTTTCCACCTCGGG 3’ |  |
| **7F** | 5’ CCGAACCAGTGGAAGTACAGGAAGAAG 3’ | 335 |
| **7R** | 5’ CTGCGGGCAATATTGGTTCATTACAGT C 3’ |  |
| **8F** | 5’ CAAGAAGGAATCCTTCGTCAGCAGG 3’ | 315 |
| **8R** | 5’ CTAGACGCTTTTGCAGTTGCTCTTCC 3’ |  |
| **9F** | 5’ CATCTACTGATGTAGCCCAGGAGGAAC 3’ | 726 |
| **9R** | 5’ GGTCTATAGGTTCATTGCAGTCCTCTGGTAC 3’ |  |
| **10F** | 5’ CACTGTCACTTCTACTGATGTGGCCC 3’ | 312 |
| **10R** | 5’ TCCAGGATATGAGCTCATACTTGTGAATATCAAAC3’ |  |
| **12F** | 5’ GATCAACGGGATCTCCACTGAGAATTCATC 3’ | 527 |
| **12R** | 5’ GTATTGGTTCTCTACAGTCCGCTGCTAC 3’ |  |
